# Wavelet-Based Demodulation of Multimode Etched Fiber Bragg Grating Refractive Index Sensor

**DOI:** 10.3390/s19010039

**Published:** 2018-12-22

**Authors:** Takhmina Ayupova, Marzhan Sypabekova, Carlo Molardi, Aliya Bekmurzayeva, Madina Shaimerdenova, Kanat Dukenbayev, Daniele Tosi

**Affiliations:** 1 National Laboratory Astana, Laboratory of Biosensors and Bioinstruments, 010000 Astana, Kazakhstan; takhmina.ayupova@nu.edu.kz (T.A.); msypabekova@nu.edu.kz (M.S.); abekmurzayeva@nu.edu.kz (A.B.); madina.shaimerdenova@nu.edu.kz (M.S.); daniele.tosi@nu.edu.kz (D.T.); 2 Nazarbayev University, School of Engineering, 010000 Astana, Kazakhstan; kdukenbayev@nu.edu.kz

**Keywords:** Evanescent field sensors, fiber Bragg grating (FBG), optical fiber sensor (OFS), refractive index sensors, wavelet density estimation

## Abstract

Etched fiber Bragg grating (EFBG)-based sensors are used as evanescent field sensors for refractive index detection. When the fiber thickness is thin and the refractive index sensitivity increases, the number of propagating modes increases, resulting in a spectral enlargement that complicates the interrogation of the sensor. In this work, we present a method to analyze the spectrum of a multimode etched fiber Bragg grating (MMEFBG) in the wavelet domain, which analyzes the amount of spectral density independently from the peak reflectivity value. The proposed interrogation method permits defining the integral of the spectral density as a novel and unconventional estimator. With respect to the conventional estimators based on wavelength shift, this estimator can better exploit the larger amount of information given by the spectral enlargement typical of multimode behavior. Results were obtained by etching an MMEFBG in hydrofluoric acid and using water/sucrose mixtures to evaluate the refractive index sensitivity, validating the interrogation method.

## 1. Introduction

Evanescent field sensors have been extensively used for the detection of refractive index variations surrounding an optical fiber [1,2,3,4,5,6,7]. Among several methods, including surface plasmon resonance [3], tapered fibers and microfibers [4], and long period grating [5], sensors based on an etched fiber Bragg grating (EFBG) have gained significant interest thanks to their properties [1,2]: ease of fabrication, since they are directly derived by standard FBGs; good sensitivity; narrow spectral occupancy; and compliance with wavelength-division multiplexing of modern sensing schemes. EFBG is an important technology for modern fiberoptic sensors, as it is the platform for building biosensors [6,7], where the surface of the sensor is functionalized with bioreceptors for biorecognition purposes.

An EFBG is obtained by chemically removing part of the cladding of a uniform FBG, thus exposing part of the field confined by the fiber to external variations [1]. The performance of EFBG sensors was analyzed in [2], showing that the refractive index sensitivity, calculated as the shift of the Bragg wavelength with respect to the outer refractive index variation [8], increases as the fiber diameter is thinned. Thus, in order to obtain larger sensitivity, thinner sensors are preferable.

However, in accordance with [9,10], for a thinner diameter of EFBG, we observe a variation of the grating effective refractive index, which results not only in a spectral shift, but also in multimode behavior. The larger number of modes results in a plurality of Bragg wavelengths, with a consequent increase of the EFBG spectral width. The current commercial high-resolution interrogators based on swept lasers and high-speed wavelength synchronization [11] exhibit strong barriers to interrogating a multimode EFBG (MMEFBG), since their peak-tracking routines [12] cannot detect the presence of multiple peaks. In addition, since the number of modes varies with the refractive index, changing the optical power spectral density, the interrogators cannot maintain a constant gain, but the gain factor appears to fluctuate during refractive index measurements.

In order to take advantage of the good sensitivity of the MMEFBG, a new interrogation method is proposed based on one-dimensional wavelet [13]. The MMEFBG spectrum is first normalized in order to remove the dependency on the dark value of the interrogator, and then processed with a one-dimensional wavelet. The results show that the evaluation of high-order density coefficients has good dependence on the refractive index, and therefore is a metric that can replace the traditional Bragg wavelength detection [12].

## 2. Modal Analysis

To exploit the evanescent field of an inscribed FBG optical fiber in order to sense the refractive index of a solution, it is necessary to reduce the thickness of the cladding in a way that significantly changes the guidance properties of the fiber. In a single mode fiber (SMF), the central wavelength of the reflection spectrum is determined by the Bragg condition so that:

(1) $$\lambda_{b}=2n_{eff}\Lambda \;,$$

where *n_eff_* represents the effective index of the fundamental mode, *λ_b_* is the Bragg central reflected wavelength, and Λ is the period of the grating. To a first approximation, in a context of multimode behavior, the number of resonant wavelengths increases according to the interaction between propagating modes and counter-propagating modes [8]:

(2) $$\lambda_{b,ij}=2\left(n_{eff,i}+n_{eff,j}\right)\Lambda \;,$$

where *λ_b,ij_* is the Bragg reflected wavelength given by the *i*th propagating mode interacting with the *j*th counter-propagating mode. Therefore, it is necessary to understand the modal content of a thinned cladding optical fiber in order to describe the behavior of the spectrum reflected by the FBG.

In a standard telecom fiber, guidance properties are fully determined by the index contrast between cladding and core, as the cladding thickness is significantly larger than the core radius. By etching the cladding, the structure can no longer be described by a two-layer model. It becomes a three-layer structure composed by the core, the residual cladding, and the solution to sense. This structure cannot be described by a simple model, so in order to show the guidance properties as a function of the residual cladding thickness, a numerical analysis based on finite element method (FEM) was performed.

For the simulation, a fiber that matches the specifications of Corning SMF-28 Ultra was used [14]. This fiber is characterized by a core diameter of 8.2 µm and a cladding diameter of 125 µm. The core and cladding refractive index values were modified, starting from the value of pure silica, to match the values of numerical aperture (NA) equal to 0.14 and group effective index equal to 1.4682 at 1550 nm. Simulations were performed considering a wavelength of 1550 nm. 

**Figure 1:**
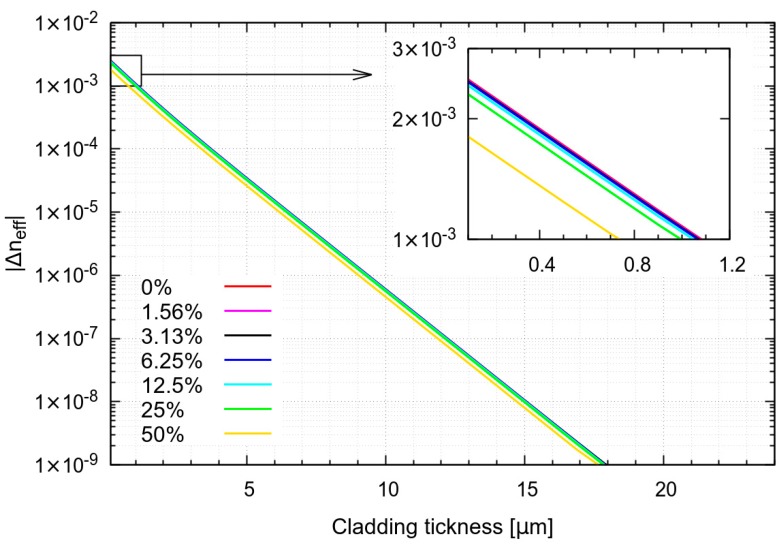
Effective index of fundamental mode with varying cladding thickness and sucrose concentration from 0% to 50% (weight/volume) in aqueous solution.

From the analysis, it is possible to notice that the effective index of the fundamental mode (FM) decreases exponentially with respect to the unetched fiber as the cladding thickness is reduced, as shown in Figure 1. The variation of effective index starts to be appreciable when the cladding thickness becomes smaller than 10 µm. The dependence on the refractive index of the solution, here described by seven solutions containing 0%, 1.5625%, 3.125%, 6.25%, 12.5%, 25%, and 50% of sucrose, where the refractive index variation is 1.85 × 10^−3^ for each 1% of sucrose increment [15], results in a shift toward smaller values of the FM effective index. This shift is small, permitting sensitivity of a few nanometers over the refractive index unit (RIU), also in the best case where the cladding is totally removed. A second effect, represented by the onset of multimode behavior, appears by reducing the cladding thickness. According to this behavior, an etched FBG would show a broadening of the reflected spectrum, permitting a new interrogation method based on wavelet demodulation.

**Figure 2:**
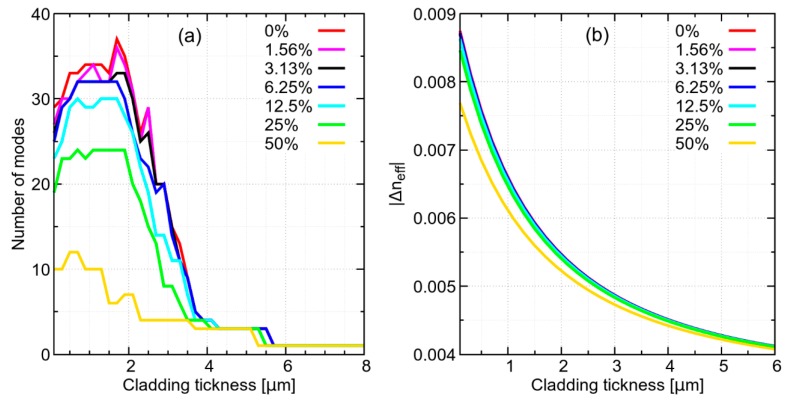
(**a**) Calculated number of modes, and (**b**) effective index distance between fundamental mode (FM) and first HOM, varying cladding thickness and sucrose concentration from 0% to 50% (weight/volume) in aqueous solution.

A three-layer structure does not permit calculating the modal content with an easy approximate formula, as with standard fiber. In particular, with standard fiber, which has a core surrounded by large cladding that can be approximated as infinite, it is possible to directly solve Maxwell equations, thus determining the cutoff condition and the number of guided modes. In a three-layer structure, obtained by reducing the cladding to a thin ring, the cladding modes are squeezed in the thin ring region. From the point of view of their effective index, which is numerically smaller than the refractive index of the cladding ring, these are cladding modes, but their intensity is spread significantly over the core. For this reason, to evaluate the number of modes, a criterion has been adopted based on the overlap integral, which is commonly used in complex microstructured fiber [16,17,18,19]. The overlap integral represents the normalized intensity that overlaps the core region, therefore it describes the fraction of mode intensity in the core. Here, a mode is considered to be a core mode when its overlap integral over the core area is larger than 0.5, so that the intensity of the mode is more confined in the core than outside the core. Using this criterion, it is possible to notice that the number of modes increases when the cladding thickness is reduced to less than 6 µm, reaching maximum discrimination around 2 µm, as show in Figure 2a. Furthermore, reducing cladding thickness improves the effective index distance (*Δn_eff_*) between modes, showing a stronger dependence of the guiding properties of higher order modes on the solution refractive index. As an example, the *Δn_eff_* between the FM and the first higher order mode (HOM) is shown in Figure 2b. It is possible to see that for small cladding thickness, *Δn_eff_* is clearly dependent on the solution properties. In conclusion, the spectral broadening dependence on the refractive index of the solution appears to be driven by two cooperating effects: variation in core mode number and variation in effective index distance between HOMs.

## 3. Setup and Fabrication

**Figure 3:**
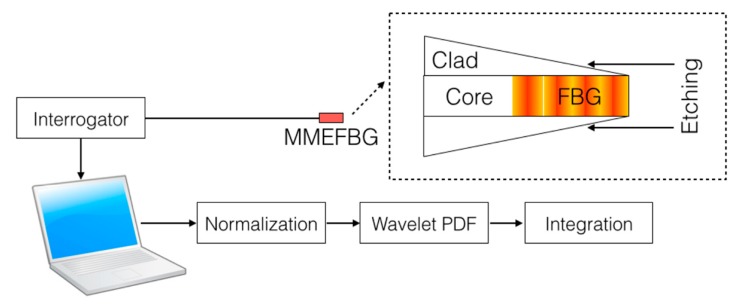
Schematic of the multimode etched fiber Bragg grating (MMEFBG) interrogation system and detection.

The setup for MMEFBG fabrication and interrogation and the process for detection are sketched in Figure 3. The experimental setup is based on a swept-laser interrogator (Micron Optics si255, 8 channel, 1460–1620 nm, 8 pm resolution, equipped with Enlight software) connected via Ethernet to a PC and returning spectral acquisition with a 1 kHz nominal rate. The wavelength resolution is 8 pm over a 160 nm bandwidth (20,000 points for wavelength grid). As with other commercial swept-laser interrogators [11], the gain of the photodetector is automatically set as a response to spectral changes, thus cannot be fixed by the user.

**Figure 4:**
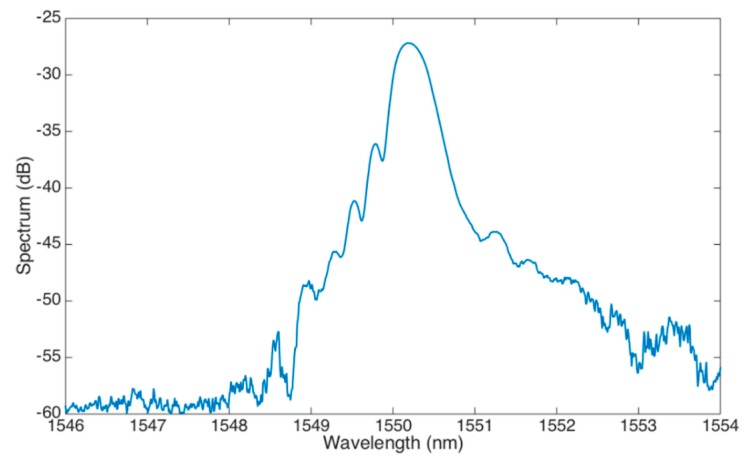
FBG spectrum before etching.

The MMEFBG is fabricated starting from a commercial FBG (Technica S.A. Atlanta, GA, USA), with 1550 nm central wavelength, 1 cm length, and 95% reflectivity, fabricated on a standard SMF-28 fiber. The reflection spectrum of the FBG before the etching is shown in Figure 4.

**Figure 5:**
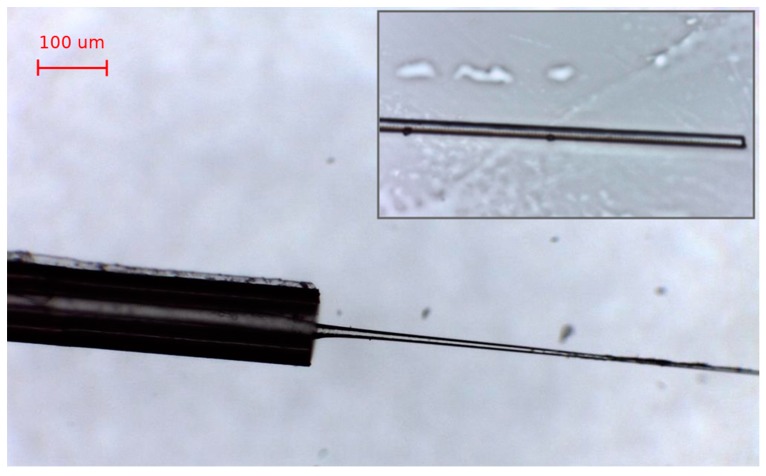
Microscope picture of the etched fiber sensor. Inset shows a detail of the sensor tip.

The fabrication and calibration for refractive index variations is performed as follows. The FBG is first cleaved on the tip side, to ensure that the etching process is enacted on the outer fiber surface. Then, the fiber is immersed in a hydrofluoric acid (HF, 48%; Sigma Aldrich) bath held at constant room temperature in a chemical fume hood (Secuflow, Waldner, ± 0.1 °C temperature stability) for 25 min. Then the outer coating is removed by dipping the fiber tip in acetone (99.8%; Sigma Aldrich) for about 1–3 min. The fiber is then inserted back into the HF solution for further etching. As in [2], the etching process removes the outer cladding, progressively exposing the FBG. The etching process has a duration of 7–14 min; the spectrum progressively shows a shift toward shorter wavelengths, as observed in [1,2], and after 7 min multiple peaks appear. In this condition, the peak tracking routine of the si255 interrogator fails, as it cannot detect the correct number of peaks. After the EFBG has been fully etched, the fiber is extracted from the HF and rinsed in water for several minutes. The sensor after the fabrication procedure is shown in Figure 5. 

The calibration to external refractive index variation was performed using a water/sucrose mixture, with the same method used in [15]. Seven solutions were prepared according to the sucrose concentration shown in the previous section. All solutions were maintained at a constant temperature (24 ± 0.1 °C) in the hood.

## 4. Interrogation and Results

The interrogation of the MMEFBG was implemented by means of wavelet signal processing [13] on a commercial signal processing toolbox (ATOMS: Scilab [20]). First, the spectrum acquired by the si255 interrogator was normalized to remove the dependence on the photodetector gain, which is arbitrarily set by the instrument. The spectra of the MMEFBG sensors are shown in Figure 4. It is possible to observe that as the sucrose concentration is increased, there is a shift toward longer wavelengths and a change of the spectral bandwidth of the FBG. In this particular grating, the spectrum appears to have a central bandwidth at the Bragg wavelength. The enlargement of the spectrum agrees with the increase in mode number. Each new mode generated by the higher index contrast of less concentrated solutions contributes to the spectrum with a smaller overlap and spectral content shifted to the left, since the higher modes present a smaller effective index. 

**Figure 6:**
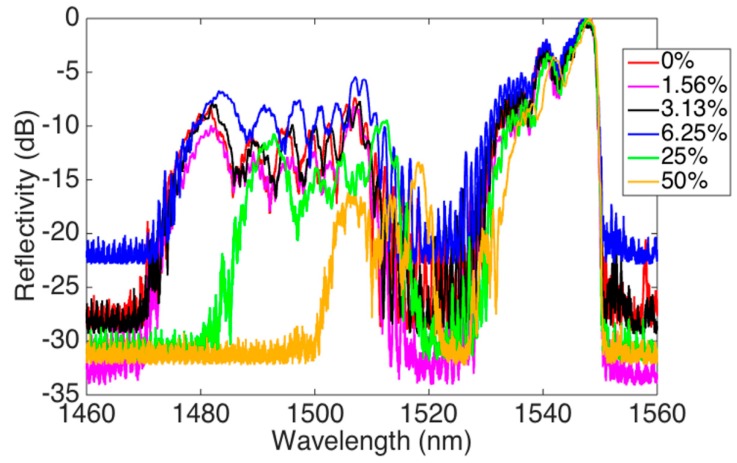
MMEFBG spectra for different sucrose concentrations.

The rationale for using wavelets rather than wavelength shift methods or correlation methods [12] is suggested by the spectral characteristic, which can be observed in Figure 6. The spectral content is not composed of a narrow bandwidth as in a pristine FBG inscribed in a standard SMF-28, but of multiple contributions, each with a different weight according to the refractive index of the analyte. Moreover, considering the fluctuation of the floor noise, the treatment of such spectra as a realization of a random variable appears to be an effective strategy, where the wavelet analysis can help to investigate the spectral density. As the refractive index increases, we observe both a significant shrinking of the bandwidth of the reflected spectrum and a shift toward larger wavelengths. The spectral features, however, appear to be noisy, in part due to the modal profile described in Section 2, in part due to synchronization of the detector in the presence of narrow spectral peaks [11]. Furthermore, since the synchronization and the peak-tracking of the device are automatically performed by whichever one chooses a suitable gain factor, the Signal to Noise Ratio (SNR) of the spectra varies for each measurement. After the normalization shown in Figure 6, this results in a fluctuation of the noise floor, apparently independent of the sucrose concentration. Performing a statistical, rather than analytical, characterization can be a way to detect the features of this noisy spectrum. In other words, if we consider the reflection spectrum of the MMEFBG displayed in Figure 6 normalized as the realization of a random variable, the wavelet one-dimensional density estimator [13,20] would be an appropriate tool to estimate its probability density function (PDF). Inferring from Figure 6, for wider spectra we would observe a larger PDF for larger reflectivity values (as there would more of them), while narrower spectra with lower high-reflectivity values would have a smaller PDF. This method, which unconventionally considers an optical spectrum as the realization of a random variable, allows a statistical analysis that moves away from the analysis of individual spectral values [12]. For this task, we use a one-dimensional wavelet analyzer with the following parameters: wavelet symlet 6 (sym6), 661 bins, soft thresholding method [20]. The computation time is approximately 10 ms.

**Figure 7:**
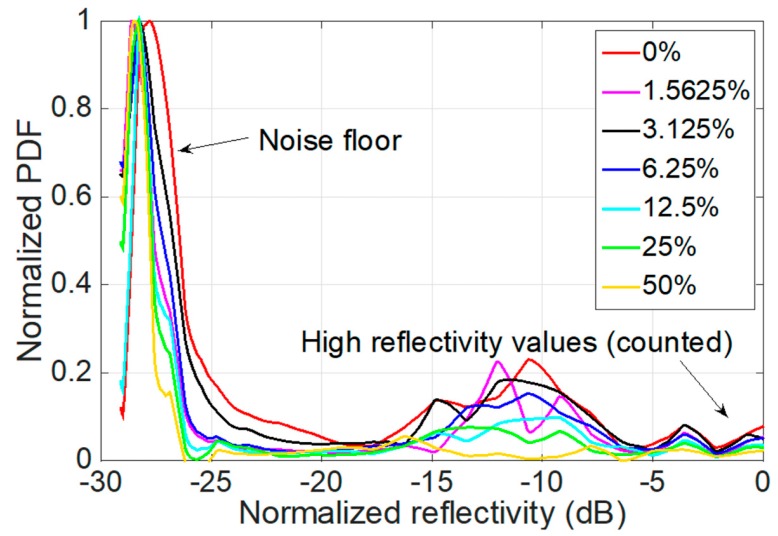
Normalized probability density function (PDF) estimated with sym6 one-dimensional wavelet estimator for each normalized MMEFBG spectral value (in dB).

The results of the wavelet density are shown in Figure 7, where the normalized reflectivity is shown in dB units. The leftmost part corresponds to the low-reflectivity values, which are the noise floor and therefore irrelevant for calculation; compared with Figure 6, these values are different for each refractive index because the interrogator noise floor is not constant, so we can disregard these data for the analysis. Conversely, the rightmost values correspond to the reflectivity peak and are accounted for in the calculation. These values correspond to the high spectral values.

A simple method to process the information in Figure 7 and return a metric for refractive index sensing, as sketched in Figure 3, is to simply integrate all normalized PDF values for reflectivity higher than −20 dB. 

**Figure 8:**
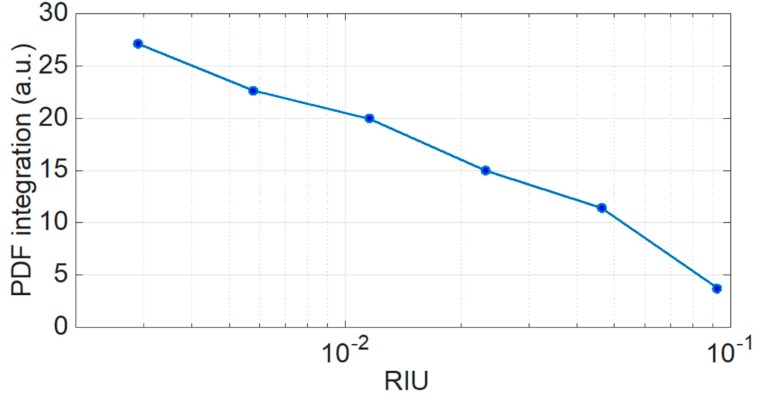
Result of integration of PDF, estimated by the wavelet operator for each variation of refractive index, reported in logarithmic units.

This final result is shown in Figure 8. For each refractive index change, the integration of the PDF (PDF_int_), as evaluated in Figure 7, shows a progressively decreasing function, varying from 29.3 for 0% sucrose (0 RIU) to 3.76 (0.09 RIU). This result, albeit obtained with an unconventional estimator, is in line with [2] and Section 2; as the outer refractive index approaches the refractive index of the cladding, the contrast is diminished and the number of modes is reduced, thus generating a visible variation of the PDF integration. It is worth noting that the usual variation of wavelength used to evaluate the refractive index is substituted by the integration of PDF estimation. Here, the sensitivity unconventionally changes with the multimode behavior of the spectrum. From Figure 8, this fact appears evident by the log scale of the refractive index variation. As a first approximation, the best sensitivity is for small sucrose concentration, where we can find a sensitivity of 1100 PDF_int_/RIU. For higher concentrations of sucrose, the sensitivity reduces to 170 PDF_int_/RIU.

The proposed method, validated in this experimental measurement, has the advantage of collecting a larger portion of information by the inherent multimodality of the MMEFBG, in particular by exploiting the spectral broadening. For this reason, it can potentially replace the traditional Bragg grating interrogation method, such as peak tracking and centroid method, mainly based on wavelength [12]. As seen in Figure 8, the method appears to be more sensitive for low values of refractive index variation, as there is a larger contrast between the outer and cladding refractive index values [1,2].

## 5. Conclusions

In conclusion, the MMEFBG fiberoptic device combines the sensitivity of refractive index variations with a multimode wideband spectrum. As traditional peak-tracking approaches fail due to the larger spectra and poor photodetector gain stability, a method based on wavelet density estimation was used. Treating the spectra as a noisy random variable, the use of wavelet permitted extracting a normalized probability density function whose noise floor is clearly detectable. By integrating the estimated PDF in an interval that cuts off the noise floor, this method is able to return a successful metric capable of tracking the spectral width and wavelength shift. In this work, a sym6 one-dimensional wavelet was used, and the high-order wavelet samples show a consistent decrease of amplitude as the refractive index increases. Data were validated experimentally. 

Future work will consist of optimizing the modal profile of the MMEFBG with respect to the wavelet interrogation and integrating the wavelet solver on board the interrogator for real-time operation.

## References

[1] Chen N., Yun B., Cui Y. (2006). Cladding mode resonances of etch-eroded fiber Bragg grating for ambient refractive index sensing. Appl. Phys. Lett..

[2] Iadicicco A., Cusano A., Cutolo A., Bernini R., Giordano M. (2004). Thinned fiber Bragg gratings as high sensitivity refractive index sensor. IEEE Photonics Technol. Lett..

[3] Sharma A.K., Jha R., Gupta B.D. (2007). Fiber-Optic Sensors Based on Surface Plasmon Resonance: A Comprehensive Review. IEEE Sens. J..

[4] Ji W.B., Liu H.H., Tjin S.C., Chow K.K., Lim A. (2012). Ultrahigh Sensitivity Refractive Index Sensor Based on Optical Microfiber. IEEE Photonics Technol. Lett..

[5] Shu X., Zhang L., Bennion I. (2002). Sensitivity characteristics of long-period fiber gratings. J. Lightw. Technol..

[6] Leung A., Shankar P.M., Mutharasan R. (2007). A review of fiber-optic biosensors. Sens. Actuators B Chem..

[7] Chiavaioli F., Baldini F., Tombelli S., Trono C., Giannetti A. (2017). Biosensing with optical fiber gratings. Nanophotonics.

[8] Erdogan T. (1997). Fiber grating spectra. J. Lightw. Technol..

[9] Kumazaki H., Yamada Y., Nakamura H., Inaba S., Hane K. (2001). Tunable wavelength filter using a Bragg grating fiber thinned by plasma etching. IEEE Photonics Technol. Lett..

[10] Lee S., Saini S.S., Jeong M. (2010). Simultaneous Measurement of Refractive Index, Temperature, and Strain Using Etched-Core Fiber Bragg Grating Sensors. IEEE Photonics Technol. Lett..

[11] Micron Optics FBG Interrogators. http://www.micronoptics.com/.

[12] Tosi D. (2017). Review and Analysis of Peak Tracking Techniques for Fiber Bragg Grating Sensors. Sensors.

[13] Donoho D.L., Johnstone I.M., Kerkyacharian G., Picard D. (1996). Density estimation by wavelet thresholding. Ann. Stat..

[14] Corning, SMF-28 Ultra. http://www.corning.com/.

[15] Shaimerdenova M., Bekmurzayeva A., Sypabekova M., Tosi D. (2017). Interrogation of coarsely sampled tilted fiber Bragg grating (TFBG) sensors with KLT. Opt. Express.

[16] Molardi C., Poli F., Rosa L., Selleri S., Cucinotta A. (2017). Mode discrimination criterion for effective differential amplification in Yb-doped fiber design for high power operation. Opt. Express.

[17] Molardi C., Sun B., Yu X., Cucinotta A., Selleri S. (2016). Polarization-Maintaining Large Mode Area Fiber Design for 2-µm Operation. IEEE Photonics Technol. Lett..

[18] Dauliat R., Gaponov D., Benoit A., Salin F., Schuster K., Jamier R., Roy P. (2013). Inner cladding microstructuration based on symmetry reduction for improvement of singlemode robustness in VLMA fiber. Opt. Express.

[19] Jørgensen M.M., Petersen S.R., Laurila M., Lægsgaard J., Alkeskjold T.T. (2012). Optimizing single mode robustness of the distributed modal filtering rod fiber amplifier. Opt. Express.

[20] ATOMS Wavelet Processing Toolbox. https://atoms.scilab.org/toolboxes/swt/.

